# Mineral water intake reduces blood pressure among subjects with low urinary magnesium and calcium levels

**DOI:** 10.1186/1471-2458-4-56

**Published:** 2004-11-30

**Authors:** Ragnar Rylander, Maurice J Arnaud

**Affiliations:** 1 Department of Environmental Medicine, Sahlgrenska Academy at Göteborg University, Gothenburg, Sweden; 2 Nestlé Water Institute and Nestlé Ltd, Vevey, Switzerland

## Abstract

**Background:**

Several previous epidemiological studies have shown a relation between drinking water quality and death in cardiovascular disease whereas others have not found such a relationship. An intervention study was undertaken to evaluate the effect of water with added magnesium and natural mineral water on blood pressure.

**Methods:**

A group of 70 subjects with borderline hypertension was recruited and consumed 1) a water low in minerals, 2) magnesium enriched water or 3) natural mineral water, in a random, double blind fashion during four weeks.

**Results:**

Among persons with an initial low excretion of magnesium or calcium in the urine, the urinary excretion of magnesium was increased in the groups consuming the two waters containing magnesium after 4 weeks. A significant decrease in blood pressure was found in the group consuming mineral water at 2 and 4 weeks.

**Conclusion:**

The results suggest that minerals taken in water are significant for the body burden and that an intake of mineral water among persons with a low urinary excretion of magnesium or calcium may decrease the blood pressure. Further studies should investigate the extent of mineral deficiency in different populations and the efficiency of different vehicles for supplying minerals, particularly magnesium and calcium.

## Background

A relation between mortality from ischaemic heart disease (IHD) and drinking water characteristics was first shown in Japan in 1957 [1]. Since then, several studies have demonstrated the same relationships, one of the last being a study from Finland in 2004 [2] and reviews have been presented [3,4]. Other studies have, however, not found such relationships or only weak associations between mineral intake and risk for cardiovascular disease [5,6]. This discrepancy may be due to an absence of causality or to variations in the populations studied regarding intake of minerals.

In a case-control study, an inverse relation was found between the amount of magnesium in drinking water and death from acute myocardial infarction and for females also between the amount of calcium and death [7]. Diets rich in vegetables and fruit, which contain high amounts of minerals, had a protective effect on cardiovascular disease [8,9]. This suggests that the mineral balance in individuals depends on different types of intakes which may vary depending on geographical and socio-economical conditions.

Regarding individual minerals, several studies have been reported where hypertensive subjects were treated orally with nutritional doses of magnesium [10]. The results suggested a dose-dependent reduction in blood pressure from the magnesium intervention but it was concluded that the relationship must be confirmed in larger studies, using higher doses of magnesium. A similar meta-analysis reported a very small effect of calcium supplementation [11]. A meta-analysis of 33 studies on potassium intervention concluded that there might be a beneficial effect on blood pressure [12]. Many of the studies reviewed were, however, dietary intervention studies, and the intervention thus comprised several minerals and other agents rather than potassium alone.

Epidemiological studies on cardiovascular disease suggest that drinking water is an important vehicle for the supply of minerals [7]. This is supported by data from short-term intervention studies using mineral water, as well as an epidemiological study [13-15].

The present intervention study was undertaken to determine the effect of minerals in water on one of the major risk factors for cardiovascular disease – blood pressure. Subjects with slightly elevated blood pressure consumed water with different levels of minerals. Serum and urinary levels of minerals were measured as a marker of intervention and blood pressure was measured before and after the intervention.

## Methods

### Subjects

Female and male subjects, aged 45 – 64 years (n = 70) were recruited by advertising in local newspapers. Inclusion criteria were living in an area with low magnesium content in the drinking water, systolic pressure 15 mm above normal values for their age, diastolic pressure above 90 mm Hg, and within 20% of ideal body weight. Exclusion criteria were hypertension target organ damage, chronic diseases (heart, liver, kidney, diabetes mellitus), pregnancy, and taking oral contraceptives or regular intake of mineral supplements. Subjects with a diastolic pressure above 100 mm Hg were advised to consult a physician for treatment. A few persons decided not to seek a physician's advice and choose to participate anyway. The Ethical committee at the Medical faculty, University of Gothenburg, approved the study.

### Blood pressure

Blood pressure was measured using standardized techniques before the intervention, at 2 weeks and at the end at 4 weeks. Two separate recordings were made (diastolic pressure as Korothoff phase 5) after 5 minutes of supine rest. The blood pressure is reported as the average of these recordings.

### Blood and urine samples

Blood samples were taken before and after the intervention to determine the serum concentration of magnesium, calcium, sodium, creatinine and potassium (analysis performed at the accredited laboratory for Clinical Chemistry, Sahlgren's Hospital, Gothenburg). Before and after the intervention period, 24 hours urine samples were collected and the amounts of magnesium, calcium, and creatinine were determined (*idem*). Magnesium and calcium levels in urine were expressed as the creatinine ratio.

### Intervention

The participants were randomly allotted into three groups to which the three waters were supplied in similar bottles marked A, B and C. The composition of the waters (see Table 1) was unknown to the persons engaged in the intervention study. The subjects were asked to consume at least one liter of water/day. When preparing coffee and tea, ordinary tap water could be used. There were no difficulties in consuming the allotted quantity and spot checks were made to control for the proper consumption. The intervention lasted 4 weeks. None of the subjects changed their normal dietary habits during the trial.

**Table 1:** Composition of the three waters used in the intervention study. ^(1)^Valvert^®^; ^(2)^Distilled water+MgSO_4_; ^(3)^Contrex^®^.

**Waters Minerals/mg L**	**A^1^**	**B^2^**	**C^3^**
Calcium (Ca^2+^)	67.6	4	486
Magnesium (Mg^2+^)	2	82.3	84
Sodium (Na^+^)	1.9	2.4	9.1
Potassium (K^+^)	0.2	0.1	3.2
Sulphate (SO_4_^2-^)	18	326	1187
Bicarbonate (HCO_3_^-^)	204	12	403
Chloride (Cl^-^)	4	0.7	8.6
Fluoride (F^-^)0	<0.05	0	0.32
Silica (SiO_2_)	5.7	0	8

### Statistical evaluation

The three groups were compared using the Student's t-test for paired samples and p < 0.05 was considered statistically significant. For a smaller subgroup of 6 individuals, comparisons were made using the Wilcoxon test for pairs.

## Results

In the analysis of the whole group, no differences in any parameters were found between persons consuming the different types of water. For the subsequent analysis, subjects with serum or urine values in excess of the 75 percentile were excluded on the ground that these represented a group with a sufficient body burden of the minerals and would not be influenced by the intervention. For magnesium in urine, this value was 0.39 mmol/l, and for calcium 0.50 mmol/L. For magnesium, calcium, potassium and sodium in blood the values were 0.9, 2.4, 4.4 and 141 mmol/L, respectively.

There was a close relation between the amount of calcium/creatinine and magnesium/creatinine in the urine before the intervention (p = 0.001). Table 2 shows the amounts of calcium and magnesium in urine before and after the intervention with different kinds of waters. It is seen that persons consuming waters B and C had significantly higher amounts of magnesium in the urine after the intervention. No significant effects of the waters on serum levels of magnesium could be detected (data not shown).

**Table 2:** Magnesium/creatinine and calcium/creatinine in urine (mmol/L) among subjects with an initial level less than 0.40 mmol/L magnesium before and after intervention with different waters.

**Water**	**n**	**Before**	**After**	**p**
**Magnesium**				
A	18	0.25 (0.08)	0.26 (0.07)	
B	18	0.28 (0.06)	0.34 (0.09)	0.009
C	19	0.30 (0.07)	0.35 (0.09)	0.019
**Calcium**				
A	18	0.40 (0.22)	0.35 (0.40)	
B	18	0.33 (0.12)	0.35 (0.40)	
C	19	0.34 (0.13)	0.38 (0.16)	

Table 3 shows the blood pressure before and after intervention. Among persons consuming water C, both the systolic and diastolic blood pressures decreased significantly at 2 and 4 weeks. A similar result was obtained when the group with an initial low level of calcium in the urine was evaluated (data not shown).

**Table 3:** Blood pressure among subjects with an initial magnesium level in urine less than 0.40 mmol/L before and after intervention with different waters. * denotes group with initial systolic values below 170 mm (see text).

**Water**	**n**	**Before**	**2 weeks**	**4 weeks**
**Systolic**				
A	18	151.9 (9.8)	154.2 (15.9)	148.3 (12.4)
B	18	148.3 (10.5)	146.8 (13.6)	147.9 (11.5)
C	19	156.8 (15.9)	150.1(16.1)p = 0.034	150.4(15.5)p = 0.017
C*	13	148.8 (13.3)	142.5(10.1)p = 0.028	144.3(14.4)p = 0.047
**Diastolic**				
A	18	90.1 (4.4)	91.2 (6.1)	89.8 (5.0)
B	18	90.4 (4.2)	89.3 (6.0)	90.9 (6.6)
C	19	91.7 (6.3)	88.0 (7.6) p = 0.014	89.1 (8.0) p = 0.020
C*	13	91.3 (6.4)	87.3 (6.3) p = 0.004	88.1 (8.6) p = 0.012

In spite of the random allocation to the different waters, it was found that the group consuming water C comprised a larger number of persons with a high initial systolic pressure. In the groups receiving waters A and B, none of the subjects had systolic blood pressures above 170 before the intervention. The subjects drinking water C were divided into those with an initial systolic pressure above and below 170 mm. For the group with the higher pressure (n = 6), there was a decrease in the systolic pressure before and at 4 weeks (p = 0.023) but no difference at 2 weeks or for diastolic pressure. The results from the remainder of the group are also shown in Table 3. It is seen that significant reductions were observed both for systolic and diastolic blood pressure after 2 and 4 weeks.

## Discussion

The study is of exploratory character, based on a relatively small number of subjects and should be interpreted with care. There is also a lack of some data that retrospectively would have been of interest such as sodium in the urine and the effect of water with only calcium added. We do not think, however, that this has any influence on the major conclusions from the study.

The intervention with the two waters with added magnesium influenced the body burden in terms of an increased excretion of magnesium in urine. This is consistent with findings from previous intervention studies [16,17] although the dose of magnesium used here was rather low in comparison to several previous studies [10]. It could have been of interest to study the effect of different doses of magnesium only, but in view of the conclusions regarding the better effect of total mineral water on blood pressure, this does not have a high priority. The absence of an effect on serum was expected; it has previously been shown that serum magnesium is a poor indicator of the body burden or the intracellular content [18].

The intervention with water containing high amounts of several minerals decreased the blood pressure significantly in contrast to water with magnesium only where no significant effect was detected. This does not exclude that an effect could have been found with the latter water, had the intervention time been longer. On the other hand, the finding supports the concept that interventions should be performed under conditions similar to the ones present in normal environments, rather than with one specific agent. This could also explain the lack of an effect in previous studies where single minerals have been given as reviewed in the introduction.

## Conclusion

In summary, the results suggest that waterborne minerals constitute a supply for the body burden, that the urinary excretion can be used as a physiologically relevant indicator of the body burden of magnesium and calcium, and that the supplementation of magnesium together with other minerals may reduce blood pressure among persons with a low body burden of magnesium and calcium, either due to an insufficient intake through food or water, or through some metabolic/clinical disturbance. Additional studies are needed to explore this further.

## Competing interests

The study was supported by an unconditional grant to Gothenburg University from the Nestlé Water Institute, Vittel, France. The authors had full freedom for data analysis, manuscript preparation and submittance to a journal. RR has not received any fees or salaries for this work, including article processing charges, nor does he own any shares or has any other financial interest in the company. MA was an employee at the Water Institute at the time of the study.

## Authors' contribution

RR and MA jointly developed the research plan. RR conducted the field study. RR and MA jointly analyzed the data and wrote the manuscript.

## Pre-publication history

The pre-publication history for this paper can be accessed here:


